# Is Green Exercise for All? A Descriptive Study of Green Exercise Habits and Promoting Factors in Adult Norwegians

**DOI:** 10.3390/ijerph13111165

**Published:** 2016-11-23

**Authors:** Giovanna Calogiuri, Grete G. Patil, Geir Aamodt

**Affiliations:** 1 Department of Dental Care and Public Health, Faculty of Public Health, Hedmark University of Applied Sciences, Hamarveien 112, Elverum 2411, Norway; 2 Section for Public Health Science, Department of Landscape Architecture and Spatial Planning, Norwegian University of Life Sciences, P.O. Box 5003, Ås NO-1432, Norway; grete.patil@nmbu.no (G.G.P.); geir.aamodt@nmbu.no (G.A.)

**Keywords:** health promotion, leisure, outdoor recreation, social inequalities, health equity

## Abstract

***Background:*** Physical activity (PA) in natural environments, known as green exercise (GE), can provide health benefits above and beyond PA in other environments, but little is known about the extent to which GE is an accessible form of weekly PA across different social groups. This study aims to examine the “GE phenomenon” in Norway, and evaluate possible differences in GE habits and perceived factors that promote GE across sub-groups of this population. ***Methods:*** 2168 adults from all over Norway reported weekly GE and other forms of PA, specific forms of GE, and perceived factors that promote GE. Data were examined in the overall sample and in relation with the respondents’ PA status and sociodemographic characteristics. ***Results:*** GE, especially “walking in the forest” and “activities by/on the sea”, was the most popular form of weekly PA, even among those with low PA levels. GE was fairly represented across all social groups, and especially among the elderly, those who live with spouse/partner and those who live in the west regions of Norway, while no associations were found in relation to sex, centrality, education level or household income. “Time flexibility” and “PA-supportive places” were generally perceived as the most important factors that promote GE across all social groups. “Accessibility to nature” was generally perceived relatively little important, though a gradient was observed in relation to age, education level and household income. ***Conclusions:*** GE is an important source of weekly PA and health among adult Norwegians, especially in sub-groups of interest for public health such as the elderly, those with lower socio-economic status and those who live in non-urban areas. More should be done to understand and address the inequities relative to the perceived accessibility to nature.

## 1. Introduction

Activities such as walking, jogging or exercising can enable people to spend time in natural environments (e.g., forests, coasts, urban green spaces, etc.), providing even greater health benefits than those related to physical activity (PA) alone [1,2]. Any PA taking place in natural environments has been defined as green exercise (GE) [3], and in the last decade studies have demonstrated the additional health benefits associated with this form of PA. For example, evidence shows that, compared with PA taking place indoors or in other built environments, GE can elicit stress reduction and positive psychological states [1,4], and provide long-lasting benefits in terms of physical and mental health [5,6]. Furthermore, it has been argued that the positive psychophysiological effects of GE could lead to greater PA adherence [4,7]. Despite the growing body of evidence regarding the relationship between GE and health, to date, little is known about the extent to which GE is a common form of PA in the general population. Moreover, questions have been raised about whether GE is a form of PA accessible to all. 

The World Health Organization (WHO) defines health equity as “the absence of avoidable or remediable differences among groups of people, whether those groups are defined socially, economically, demographically, or geographically” [8]. Such differences, or inequities, often relates to access to treatment, health outcomes and quality of life, but they have also been described in relation to PA participation [9] as well as the extent to which people visit natural environments and use them for PA purposes [10,11]. For example, it has been shown that in Norway PA levels increases with increasing education level and, to a lesser extent, household income [12]. As remarked in a literature review of European studies, low income groups might especially meet challenges in engaging in forms of leisure-time PA that require payment of memberships or expensive equipment, but also because these groups tend to live in areas with poor availability of safe and attractive places where one can engage in PA [9]. Levels of PA are commonly reduced among the elderly, thought this effect was found to be attenuated in older adults living with their spouse/partner [12,13]. Geographical characteristics can also influence the extent to which people engage in regular PA. Rural residents have been generally found to be less active than urban residents, a phenomenon partly explained by a reduced engagement in activities such as walking or biking for transportation [11]. 

Common characteristics of social groups that experience health inequities are reduced political, social or economic power [8]. However, when it comes to PA participation, the physical and perceived environment plays a central role [14]. Especially, to engage in GE, it is important that people perceive having access to safe and attractive natural environments that can support PA [15,16]. However, a systematic review of the literature emphasized that the extent to which people use available natural environments for PA purposes can also be influenced by individual determinants such as age, sex and ethnicity [10]. Furthermore, some forms of GE like hiking in the mountains, climbing, canoeing or other outdoor recreations in remote places can be limited by personal barriers [17,18]. Such activities require in fact resources like personal skills, specific equipment, and the possibility to travel to particular locations, characteristics that are more common among those with higher self-efficacy for PA, as well as higher socioeconomic status [9]. It would be therefore expected that some sub-groups of the population, like those with lower PA profile and those with lower socio-economic status, are less engaged with GE than others. Because of safety perceptions, women and the elderly might also be less prone to engage in GE. 

In Norway and other Scandinavian countries, many people consider activities in close contact with nature such as hiking, skiing and fishing, a tradition, especially during holiday periods [17]. Moreover, in Norway, many people live in the vicinity of natural environments, and this, alongside the lively culture of outdoor recreations, might encourage people to engage in “low threshold” forms of GE like walking or jogging in nearby areas. This might have positive impact in terms of equity in PA participation. National surveys in the Norwegian population seem to corroborate such assumption. For example, it was found that in 2011, 76% of adult Norwegians engaged in GE at least once a month, as opposed to 46% of adult Norwegian who exercise in the gym at least once a month [19]. Short walks in the forest appear to be especially popular in Norway, as 79% of adults reported to have engaged in this activity at least once in the past year [13]. Some analyses also indicate that GE is less subjected to inequities as compared to organized exercise in the gym: it was in fact found that while among those who engaged in the gym- and sport-based PA there was a larger proportion of individuals with higher socio-economic status, such pattern was not found among those who engaged in GE [19]. However, all these studies refer to activities that might occur *occasionally* (i.e., “at least once in a month” or “during the past year”), whereas in order to understand the contribution of GE in in terms of health enhancing PA, one should rather look at activities taking place on a *weekly* base. Health institutions such as the WHO and the Norwegian Directorate of Health recommend in fact that adults engage in relatively high amounts of PA during a regular week (i.e., at least 150 min/week of moderate-intensity aerobic PA, or 75 min/week of vigorous-intensity aerobic PA) [20]. 

In light of the above premises, the purpose of this descriptive study is two-fold: (a) to provide a comprehensive overview of the “GE phenomenon” in Norway by examining to what extent weekly GE, in different forms, is a common source of weekly PA among adult Norwegians; and (b) to evaluate the significance of GE to health equity, examining the distribution of weekly GE and possible factors that promote GE across different social groups. Throughout this study, we are particularly interested in examining patterns of weekly GE among individuals with low physical activity levels, as well as the impact of possible social determinants such as sex, age, socio-economic status, family status and geographical characteristics.

## 2. Materials and Methods

### 2.1. Design and Respondents

In 2012, *Norsk Friluftsliv* initiated a national cross-sectional survey that aimed to explore PA habits and motivational factors among Norwegians, with attention given to GE and outdoor recreations. A web-based survey was carried out by a Norwegian marketing agency (Ipsos MMI), during October 2012. An invitation to participate was sent to 8620 individuals aged 18 or older randomly selected from a panel of approximately 50,000 individuals who regularly participate in their surveys. The sampling process was stratified by sex, age and geographical area in order to reduce possible selection bias and so that the final sample would be representative for the Norwegian population. The invitation was sent via e-mail, and one reminder was sent to those who did not respond to the first invitation. The final dataset contains data from 2168 respondent (response rate = 25%). In this paper, only variables of interest for the study purposes will be presented and discussed, whereas more details are reported elsewhere [21]. 

### 2.2. Independent Variables

*Physical activity status*: Overall moderate- to vigorous-intensity physical activity (MVPA) was measured by an item inquiring “For how much time (hours and minutes), through the course of a regular week, do you engage in activities that increase your breathing or make you sweat?” The item was provided with explanatory examples (“This can include, for example, exercising/training, occupational activities or activities at school, walking/hiking in the forest, and when you walk or bike to and from work or school”). This continuous variable was used to divide the respondents in three *PA status groups* in line with WHO’s recommendations for moderate-intensity aerobic PA, which are the same for all adults aged 18 or older, including those aged ≥65 years [20]: (i) Low PA levels (LA) = respondents with total PA being less than 150 min/week; (ii) Recommended PA levels (RA) = respondents with total PA being between 150 and 299 min/week; and (iii) High PA levels (HA) = respondents with total PA being more 300 min/week. 

*Sociodemographic characteristics*: The sociodemographic items covered age, sex, socio-economic status, living situation, and geographical information. Indicators of socio-economic status were yearly household income and educational level. Family status consisted information about living situation (i.e., whether one lives alone, with spouse/partner, with their parents or with friends), and whether or not there were small children in the household. Geographical information included region of residence, and centrality (whether one lives in a large city, small city, small town/village, or in the countryside). The different regions were grouped in seven major geographical areas: Oslo and Akershus (eastern regions), Hedmark and Oppland (east-inner regions), South Eastern Norway, Agder and Rogaland (south-western regions), Western Norway, Trøndelag (mid region), and Northern Norway. To be noted that the three largest cities of Norway, Oslo, Bergen and Trondheim, are located in the regions of Oslo and Akershus, Western Norway and Trøndelag, respectively. All information was self-reported but centrality and region, which were automatically determined based on the respondents’ ZIP-codes. For all questions, alternative response options such as “I don’t know”, “I can’t answer” or “It does not apply to me” were included, in order to avoid possible misleading or missing answers. 

### 2.3. Dependent Variables

*Weekly green exercise*: The questionnaire included a list of specific forms of PA, and the respondents were asked to report whether they spent any time in each of them within a regular week. One of these activities was “walking or exercising in parks, green spaces or other natural environments”, which was defined as “green exercise” for the purpose of this study. Although GE was the variable of main interest for the purposed of this study, the other forms of PA were also used as a comparison. In this way we could understand how popular GE is as compared with the other forms of PA, which included: “exercising in the gym”, “participation in organized sports”, “active transport to and from work/school”, “walking the dog or other companion animal”, and “occupational or school-based PA”. The respondents were also asked to report the amount of time (minutes per week) they spent in each activity. As the list of activities was not comprehensive (e.g., activities such as dancing or walking in the city were not listed), an additional variable “other” was created by subtracting the time spent in the above listed forms of PA from the overall amounts of weekly MVPA.

It should be noted that, given possible overlaps with other activities listed, our measure of GE shall be intended as a form of leisure-time PA, which was perceived by the respondent qualitatively different than all other forms of PA. For instance, this measure of GE excluded activities of more instrumental nature that may take place in natural environments, e.g., walking the dog in a neighborhood park or commuting to work by walking or cycling through a green area. Such a restrictive definition of GE was applied because in the questionnaire it was not specifically stated whether or not these forms of PA took place in natural environments. For example, while walking the dog, active transport and even sports might be performed in contact with nature, they might as well be performed in urban settings or other built environments. To avoid possible overestimates of GE, it was therefore preferred to exclude such activities. 

*Specific forms of green exercise*: The extent to which the respondents frequently engaged in specific forms of GE was measured by eight items, consisting in statements like: “I often walk/hike in the forest” or “I often engage in activities by/on the sea”. Among these statements, there was one stating “I often cycle”, which could relate to GE as well as transportation PA in urban settings or other built environments; this item was retained for further analysis to get some understanding of the impact of this form of locomotion as a potential source of GE experiences, but it should be interpreted with caution in terms of actual form of GE. An additional statement, “I often experience nature quietness”, referred to potentially restorative experiences of nature that might or might not be associated with PA. Despite this item did not necessarily relate to PA, it was included to estimate the extent to which the respondents perceived to be frequently exposed to potentially restorative experiences of nature. 

For all items, the respondents reported on a scale from 1 to 4 (1 = “it does not fit me”; 4 = “it fits me very well”) indicating how well the respondent perceived the statements to describe themselves. An option “not applicable” was also included, but excluded from further analysis. Considering the fact that all statements made explicit reference to engaging in a given activity “often”, the extent to which the respondents agreed with each statement should be intended as a measure of frequency. For example, if one responded “it does not fit me” to the statement “I often walk/hike in the forest”, it is fair to infer that that person seldom walk/hike in the forest. It should be noted however that such measure reflects a subjectively perceived dimension, as in fact no objective parameter of frequency (e.g., “every week” or “every day”) was provided. To simplify the analysis and interpretation of the findings, the rating of all items were dichotomized and re-named as 0 = “never/seldom” (which comprises the original ratings 1 = “it does not fit me” and 2 = “it fits me little”) and 1 = “fairly frequently” (which comprises the original ratings 3 = “it fits me well” and 4 = “it fits me very well”).

*Perceived factors that promote green exercise*: Through a multiple choice item, the respondents reported whether they would engage more in GE (expressed as “engaging in PA outdoors in contact with nature”) in the future. The item included also other answer alternatives, which were “exercising in the gym”, “participating in indoor or outdoor sports”, “other”, and “I don’t intend to engage in more PA”. Only the respondents who reported future intent for GE (*n* = 1369) were used for further analysis, while all the respondents who selected “I don’t intend to be more active” or other forms of PA were excluded. It should be noted that the respondents could select only one answer alternative, therefore their response should be considered as their “first choice”, or preference. The respondents were then presented with a set of 18 statements related to factors that would help them becoming more physically active. These included statements such as “being invited by friends” or “better parking opportunities close to parks, green spaces and natural environments”. The statements were rated on a 4-point scale of perceived importance (1 = “Not important”; 4 = “Very important”). An option “not applicable” was also included, but excluded from further analysis. The perceived importance the respondents assigned to each of these items was therefore regarded as possible factors that promote more engagement in GE. A factor analysis, using Principal Component Analysis, was performed based on items loading of at least 0.45 and eigenvalues greater than 1.0. Identified components were given a short descriptive name, and mean scores were calculated based on the score of the included items. Four major components were identified, which were named “accessibility to nature”, “Social support”, “PA-supportive places”, and “institutional support”. Two items that specifically related to indoor exercise facilities (i.e., “better access to fitness centres” and “… swimming pools”) were excluded in this study. One item, representing “Time flexibility” (i.e., “That … I can do it when it suits me better”) was also excluded from the four major components because of small factor loading, but was used as individual item in further analysis. A series of Spearman’s rank correlations showed that all the factors that promote GE were significantly and positively correlated with each other. Especially, rho (ρ) coefficients of moderate magnitude were observed between “accessibility to nature” and both “PA-supportive places” (ρ = 0.63; *p* < 0.001) and “institutional support” (ρ = 0.40, *p* < 0.001). All other correlations were of weak magnitude, with rho ranging between 0.22 and 0.34, and especially “time flexibility” showed quite weak correlations with “PA-supportive places” (ρ = 0.17; *p* < 0.001) and “institutional support” (ρ = 0.16; *p* < 0.001). The emerged categories (ranked by eigenvalue) with included items are presented in Table 1. 

### 2.4. Statistical Analysis

First, to provide an overview of the “GE phenomenon” in Norway, we examined the prevalence of people engaging in any weekly GE (i.e., those who engaged in GE for ≥1 min/week) as opposed to the other forms of PA. To better understand the PA profile of those who engage in GE, this was done in the overall sample and stratifying by PA status. A Chi-squared (χ^2^) test for independence was used to establish possible associations of the respondents’ PA status with engaging in weekly GE and the different forms of PA during a regular week. We then defined four levels of GE by re-coding the variable “weekly GE” as follow: (i) 0 min/week; (ii) 1–59 min/week; (iii) 60–149 min/week; and (iv) ≥150 min/week. These levels help us understanding how many respondents engaged in weekly amounts of GE that can substantially contribute to achieving health enhancing amounts of PA. For example, the cut-off of ≥150 min/week grossly corresponds to the minimum recommended levels of moderate-intensity aerobic PA, whereas the cut-off of 60–149 min/week represents a substantial amount of PA, covering a large part of such recommendations. Furthermore, in order to examine to what extent the levels of GE were associated with potentially restorative experiences of nature, a χ^2^ test for independence was used to establish possible associations of the four levels of GE (i.e., 0 min/week, 1–59 min/week, 60–149 min/week and ≥150 min/week) with “experiencing nature’s quietness”. Eventually, to examine more in depth what the respondents’ GE habits were and the way such habits related to their PA levels, we examined the proportion of the different specific forms of GE, as well as across the different PA status groups. A χ^2^ test for independence was used to establish possible associations of the respondents’ PA status with the different forms of GE. 

To evaluate the significance of GE in terms of health equity, the prevalence of weekly GE (i.e., whether or not one engages in GE for ≥1 min/week) was examined across the different socio-demographic groups, to assess how this form of PA was distributed in the population. Logistic regression was used to establish possible associations of weekly GE with the different sociodemographic variables (tested individually). We then investigated possible patterns relative to the perceived factors that promote GE. To do so, we selected only the respondents who reported future intent for GE (*n* = 1369), while all the respondents who selected “I don’t intend to be more active” or other forms of PA were excluded. A multivariate analysis of variance (MANOVA), using Wilks’ Lambda (λ) as a parameter, was then used to establish whether there was an effect of PA status, weekly GE, and the different sociodemographic characteristics (individually set as between-subjects factor) on the perceived factors that promote GE. If a significant multivariate effect was observed in relation with a given independent variable, a univariate analysis of variance (ANOVA) was performed to establish possible effects on the individual dependent variables. 

In order to examine more in depth how GE habits and perceived factors that promote GE are distributed across different social groups, the analyses described above were performed stratifying the sample for different socio-demographic characteristics. Although the findings of these analyses do not directly address the main purpose of this study, they provide additional information about the “GE phenomenon” in Norway and its value in terms of equity in PA participation across different social groups of interest in public health. These findings are not presented in the main text of this paper, although those of major interest are presented in Supplementary Material. Such Supplementary Material includes two series of images depicting the relative prevalence of respondents who engage in weekly GE or other forms of PA (Figure S1a–h) and those who reported to engaged in different forms of GE “fairly often” (Figure S2a–h) stratified by all the socio-demographic variables: (a) sex; (b) age group; (c) education level; (d) household income; (e) living situation; (f) whether or not there are young children in the household; (g) centrality; and (h) region of residence. Moreover, the outcomes of the logistic regressions and the MANOVA analyses restricted to women (*n* = 1076) and older adults (age ≥ 65 years; *n* = 456) are presented in four additional tables (Tables S1–S4). Corresponding tables for men and the other age groups are not presented to avoid redundancies.

All analyses were performed using IBM SPSS Statistics version 20.0 (Chicago, IL, USA). Statistical significance was assumed with *p* < 0.05.

## 3. Results

### 3.1. Sample Description

Details about the respondents’ sociodemographic characteristics are presented in Table 2. The sample was provided a fairly representative of the Norwegian population with respect to sex, age, and geographic distribution. Men and women were well balanced across all age-groups. The middle-aged adults (45–64 years) represented the largest part of the sample (49%), while 30% of the sample consisted of younger adults (18–44 years) and 21% of the sample consisted in older adults (≥65 years)—the overall prevalence of individuals aged ≥80 accounted for 1%. Sociodemographic characteristics such as the living situation and the prevalence of people having responsibility for small children also appear fairly in line with national statistics [22]. The indicators of socio-economic status appear however slightly inflated compared to national figures. Especially, when excluding those who responded “currently studying”, in our sample only 10% of respondents had education level below upper secondary education, compared to 27% in the Norwegian population. Further, in our sample, 56% of respondents had higher education, compared to 36% in the Norwegian population [23]. It should be noted however that some methodological factors might partly explain this gap: first, national figures include individuals aged 16 years or older, whereas our sample includes individuals aged 18 years or older; secondly, the national figures include an estimated level of education for missing values for many individuals with immigration background; finally, the national figures refer to *completed* educational level, whereas our measure might have also covered incomplete educational path. Most respondents had fairly high levels of PA, with 31% reporting “recommended” PA levels (RA group, i.e., they reported to engage in MVPA between 150 and 299 min a week) and 30% reporting “high” PA levels (HA, i.e., they reported to engage in MVPA for 300 min per week or more). The remaining 40% of respondents reported “low” PA levels (LA group). Such PA levels also appear to be fairly in line with other figures on self-reported PA in the Norwegian population [12].

### 3.2. Weekly Ratings and Specific Forms of Green Exercise

**Figure 1:**
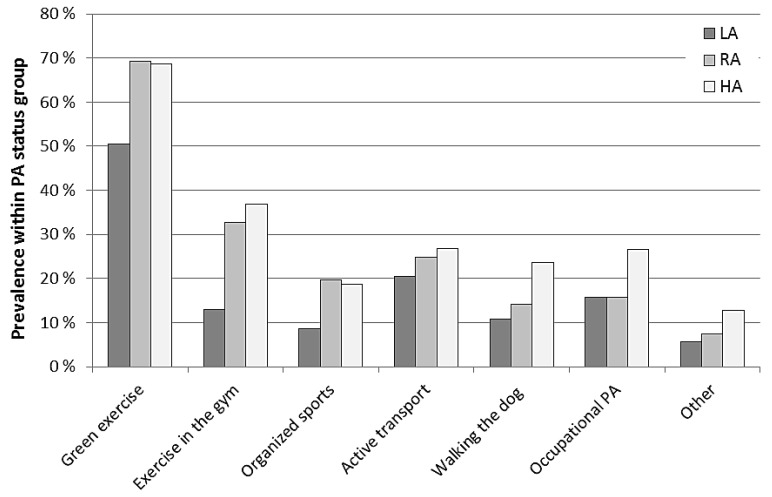
Prevalence of adult Norwegians with different physical activity status engaging in weekly green exercise and other forms of physical activity (*n* = 2168). LA = Low PA levels (MVPA < 150 min/week); RA = Recommended PA levels (MVPA = 150–299 min/week); HA = High PA levels (MVPA ≥ 300 min/week). The bars represent the prevalence of individuals who reported to engage in green exercise or other forms of PA for ≥1 min/week, whereas those who reported not to engage (=0 min/week) are not reported because it would be redundant.

In the overall sample, the prevalence of individuals who engaged in GE weekly was 62%, which was substantially larger as compared with all other forms of leisure-time PA, such as exercising in the gym (26%) and participating in organized sports (15%). The prevalence of weekly GE was also quite large as compared to the prevalence of other “instrumental” forms of PA, such as active transport (24%), occupational PA (19%) and walking/exercising with dog (16%). Unsurprisingly, all forms of PA were significantly associated with the respondents’ PA status, showing that a larger prevalence of respondents with higher PA levels (RA and HA) engaged in each form of PA during a regular week as compared with the LA respondents. Especially, gym-based exercise had the strongest associations with the respondents PA status (χ^2^ = 131.58; *p* < 0.001), followed by GE and organized sports (χ^2^ = 74.44 and 46.42, respectively; *p* < 0.001 for both). Walking the dog, occupational PA and “other” showed somewhat smaller associations with the respondents’ PA status (χ^2^ = 47.23, 34.66 and 25.22, respectively; *p* < 0.001 for all). Active transport had the smallest association among all the different forms of PA (χ^2^ = 9.03; *p* = 0.011). Despite the highly significant association of weekly GE with the respondents’ PA status, GE was the most represented form of PA in the LA group, with 51% of LA respondents engaging in some amounts of GE during a regular week (Figure 1). For the LA group, GE was far more common than other types of activities often believed to be important sources of weekly MVPA in less active individuals such as active transport (20%) and walking the dog (11%). The prevalence of LA respondents who engaged in weekly GE was also noticeably larger than the prevalence of LA respondents who exercised in the gym (13%) or participated in organized sports during a regular week (9%). Additional information about the extent to which different social groups engaged in weekly GE and other forms of PA is presented in Supplementary Material (Figure S1a–h).

Not only was GE the most popular form of PA in our sample, we also found that most of the “green exercisers” engaged in this activity in fairly high levels: only 11% of respondents engaged in GE in relatively little amounts (1–59 min/week), while the remaining 51% engaged in GE for one hour or more during a regular week (30% engaged in GE for 60–149 min/week and 21% engaged in GE for ≥150 min/week). It was also found that the majority of respondents (57%) reported to experience nature’s quietness “fairly often”, which was unsurprisingly significantly associated with higher levels of GE (χ^2^ = 140.00; *p* < 0.001): the proportion of respondents reporting to experience nature’s quietness “fairly often” was 45%, 62%, and 78% for those who engaged in GE for 1–59 min/week, 60–149 min/week, and ≥150 min/week level, respectively. It was, however, noticed that almost half (46%) of those who do not engage in GE during a regular week reported to experience nature’s quietness “fairly often”. Experiencing nature’s quietness was also associated with the respondents’ PA status (χ^2^ = 116.70; *p* < 0.001), with the proportion of respondents reporting to experience nature’s quietness “fairly often” being 44%, 62%, and 71% among the LA, RA, and HA, respectively.

**Figure 2:**
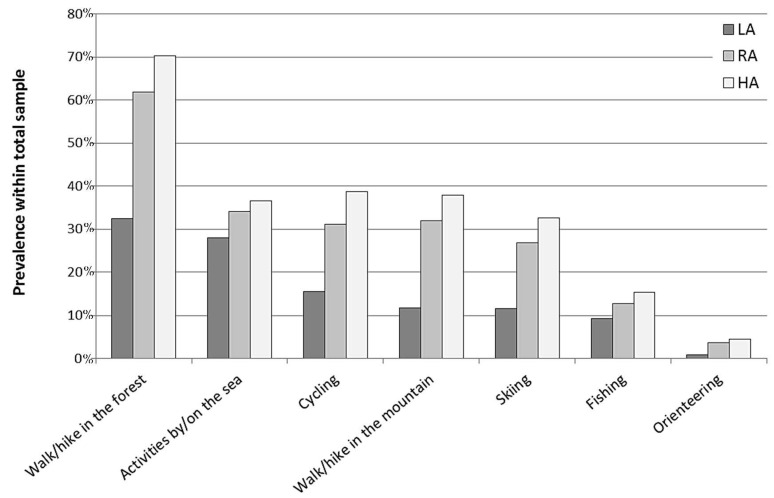
Prevalence of adult Norwegians with different physical activity status engaging in specific forms of green exercise “fairly often” (*n* = 2168). LA = Low PA levels (MVPA < 150 min/week); RA = Recommended PA levels (MVPA = 150–299 min/week); HA = High PA levels (MVPA ≥ 300 min/week). The bars represent the prevalence of individuals who reported to engage in the different forms of GE “fairly often”, whereas those who reported “never/seldom” are not reported because it would be redundant.

As shown in Figure 2, “walking/hiking in the forest” was the most frequent form of GE, as 53% of respondents engaged in this activity “fairly often”. “Activities by/on the sea” and “cycling” were also frequently endorsed activities (32% and 27%, respectively), followed by “walking/hiking in the mountains” (26%), “skiing” (23%), and “fishing” (12%). Only a small prevalence of respondents (3%) reported to frequently engage in “orienteering”, a sport in which participants navigate from point to point using map and compass, normally moving at speed and typically takes place in forests or other natural environments. Unsurprisingly, all specific forms of GE were significantly associated with the respondents’ PA status, showing that a larger prevalence of respondents with higher PA levels (RA and HA) engaged in the different forms of GE “fairly often” as compared with the LA respondents. Especially, “walking/hiking in the forest” and “walking/hiking in the mountains” had the strongest associations with the respondents PA status (χ^2^ = 243.04 and 149.91, respectively; *p* < 0.001 for both). “Cycling” and “skiing” also showed large associations with the respondents’ PA status (χ^2^ = 106.46 and 103.15, respectively; *p* < 0.001 for both), whereas the association of “activities by/on the sea”, “fishing” and “orienteering” with the PA status were somewhat smaller (χ^2^ = 14.09, 13.02 and 18.78, respectively; *p* = 0.001 for all). Despite being strongly associated with the respondents’ PA status, “walking/hiking in the forest” was the most frequently endorsed form of GE in the LA group (32%), closely followed by “activities by/on the sea” (28%). On the other hand, “cycling”, “walking/hiking in the mountains” and “skiing” proved to be little represented among the LA group (15%, 12% and 12%, respectively). Despite their small associations with the PA status, “fishing” and, especially, “orienteering” were also little represented in the LA group (9% and 1%, respectively). Additional information about the extent to which different social groups engaged in the different forms of GE is presented as Supplementary Material (Figure S2a–h).

### 3.3. Weekly Green Exercise and Perceived Promoting Factors across Sociodemographic Groups

The prevalence of weekly GE in relation to the respondents’ PA status and sociodemographic characteristics, with the corresponding odd ratios (OR) and 95% confidence intervals (CI), is reported in Table 2. Weekly GE was not evenly distributed across the socio-demographic groups. Specifically, logistic regression revealed that the prevalence of weekly GE increased age, and it was higher among those living with a spouse/partner as compared with those who live alone and those who live with friends/parents. Moreover, the prevalence of weekly GE was higher among those living in western regions of Norway (Agder/Rogaland and Western Norway), with Oslo/Akershus region as a reference. Sex, education, household income, having responsibility for small children, and centrality were not associated with weekly GE. The outcomes of the logistic regression were fairly parallel for men and women, as one may notice in Table S1. Some differences could be however noticed when the analysis were stratified by age group. Especially, as shown in Table S2, among the elderly there was no significant association of engaging in weekly GE with living situation or region. On the other hand, a significant association was found with centrality, with the older adults living in the countryside being significantly more likely to engage in weekly GE as compared with those who live in large cities.

It is interesting to note that, while GE was positively associated with older age, opposite patterns were observed for other forms of PA. Especially, the relative prevalence of older adults engaging in weekly GE was higher than the relative prevalence of younger adults (Figure S1b), while it was the opposite for almost all other forms of PA. Logistic regressions even showed that, compared with younger adults, elder adults were less likely to engage in gym-based exercise (OR = 0.61; 95% CI = 0.47–0.80; *p* < 0.001), sports (OR = 0.55; 95% CI = 0.39–0.76; *p* < 0.001) and active transport (OR = 0.14; 95% CI = 0.09–0.21; *p* < 0.001), as well as occupational PA (OR = 0.37; 95% CI = 0.26–0.53; *p* < 0.001). It should also be noted that, while we found no significant association of weekly GE with the respondents’ education level, as shown in Figure S1c, a social gradient could be observed for gym-based exercise, active transport and, to a lesser extent, organized sports. This was confirmed by logistic regressions showing that those with higher education were more likely to engage in gym-based exercise (OR = 1.95; 95% CI = 1.29–2.95; *p* = 0.002) and active transport (OR = 1.85; 95% CI = 1.20–2.85; *p* = 0.006) as compared with those who had education level below upper secondary school. Furthermore, those who were currently studying were more likely to engage in gym-based exercise (OR = 2.85; 95% CI = 1.82–4.44; *p* < 0.001), organized sports (OR = 1.73; 95% CI = 1.00–2.99; *p* = 0.049), and active transport (OR = 2.94; 95% CI = 1.85–4.65; *p* < 0.001) as compared with those who had education level below upper secondary school. Moreover, unlikely weekly GE, exercising in the gym and engaging in active transport for ≥1 min/week were also significantly associated with centrality (Figure S1g), showing a clear dose-response pattern: the magnitude of the association was in fact relatively small in the comparison of small-city dwellers with large-city dwellers (exercise in the gym: OR = 0.64; 95% CI = 0.50–0.82; *p* < 0.001; active transport: OR = 0.70; 95% CI = 0.54–0.90; *p* = 0.006), it was of intermediate size in the comparison of small-towns dwellers with large-city dwellers (exercise in the gym: OR = 0.54; 95% CI = 0.42–0.69; *p* < 0.001; active transport: OR = 0.60; 95% CI = 0.46–0.78; *p* < 0.001) and it was relatively large in the comparison of countryside dwellers with large-city dwellers (exercise in the gym: OR = 0.42; 95% CI = 0.31–0.58; *p* < 0.001; active transport: OR = 0.36; 95% CI = 0.25–0.51).

A large part of the respondents (63%) reported they would like to engage more in GE—this is the sub-sample of interest for our analysis of the perceived factors that promote GE. Many of these individuals had low PA status (LA = 40%) and did not engage in any weekly GE (34%), indicating the existence of some barriers hindering or limiting the engagement in GE in a relatively large part of the sample. As shown in Table 3, “Time flexibility” had in general the highest mean rating-score across all social groups, indicating that this was perceived as the most important factors that promote GE by most respondents. This was followed by “PA-supportive places”, whereas “accessibility to nature” had in almost all cases the lowest mean rating-score. The MANOVA showed however some differences across the different social groups (Table 3). Specifically, significant relationship of the perceived barriers were found with PA status, sex, age, education, household income, living situation and region of residence, whereas no significant relationship was found with weekly GE, having small children and centrality. 

The ANOVA found that PA status was significantly associated only with “PA-supportive places”, with the ratings of this perceived factor that promote GE increasing with increasing PA status. As compared to men, women assigned significantly higher ratings to all the perceived factors but “PA-supportive places”. A significant relationship of age was found with “accessibility to nature”, “social support”, and “institutional support”: the ratings of “social support” decreased with growing age, whereas the ratings of “accessibility to nature” and “institutional support” increased with growing age. The differences were such that for older adults “accessibility to nature” and “institutional support” were reported as more important factors than “social support”, whereas for younger adults “social support” was a more important factor than “PA-supportive places”. With respect to the latter relationship, a similar pattern was observed also for those who live with parents or friends, which is likely to be linked to the age of this social group. Education and household income were significantly associated with “accessibility to nature”, with those with lower education and income assigning greater importance to this factor than those with higher education and income. To be noted that the relationship of education with “accessibility to nature” was such that for those in the lowest education group, this factor had the third highest rating-score. Those with lower education also assigned greater importance to “institutional support”, as compared with those with higher education, and also assigned to this factor greater importance than they assigned to “social support”. Finally, living in different regions of Norway was significantly associated with giving importance to “social support”: those who live in Agder/Rogaland and Northern Norway, among the different regions, assigned highest importance to this factor.

As one may deduce from Table S3, besides the fact that women generally tended to assign higher ratings of importance to all factors that promote GE, fairly parallel patterns were found for men and women, though not in all cases the associations achieved statistical significance. When the analyses where stratified by age group, some differences were observed in the extent to which adults of different age and with different PA profiles perceived that “PA supportive places” was and important factor that promote GE: while in the general population the importance assigned to this factor increased with increasing PA status, such association was not found when looking at the older adults only (Table S4). Besides this, the findings showed fairly parallel patterns.

## 4. Discussion

The purpose of this study was two-fold: (a) to examine to what extent weekly GE, in different forms, is a common source of weekly PA among Norwegian adults; and (b) to examine the distribution of weekly GE and possible promoting factors across different social groups. Throughout the study we were especially interested in sub-groups of particular interest for public health and PA promotion, such as those with low PA levels, the elderly and those with lower socio-economic status. Family status and geographical characteristics were also possible determinants of weekly GE examined in this study.

### 4.1. Green Exercise Habits in Adult Norwegians

We found that compared with other forms of weekly PA, GE is the most popular among adult Norwegians. In fact, in the overall sample, the prevalence of respondents engaging in weekly GE was more than two-times as large as the prevalence of respondents engaging in active transport and exercising in the gym during a regular week. This finding not only confirms previous figures [13,19], but it further demonstrate that, in Norway, GE is very popular also on a weekly base, representing therefore an important source of health-enhancing PA. This is corroborated by the fact that GE is highly endorsed by those with higher PA levels (RA and HA groups), and most of the green exercisers engaged in relevantly large amounts of GE (e.g., one hour or more during a regular week). Noticeably, GE is also quite popular among those who have low PA levels (the LA group), which represent a target group of particular importance for PA promotion. In this group, the prevalence of weekly GE was more than two-times as large as the prevalence of active transport and four-times as large as the prevalence of exercising in the gym. This suggests that GE is particularly appealing to people with low PA profile, and might provide a suitable arena for enhancing their PA levels. This would be also corroborated by the large prevalence of individuals with low PA levels who reported they would like to engage more in GE.

We found that walking/hiking in the forest and activities by/on the sea are the most popular forms of GE, and were highly endorsed in the overall sample as well as in the LA group: this indicates that these activities represent “law- threshold” and easily accessible forms of PA that can be integrated in everyday routines. Activities by/on the sea might be especially important in this perspective. A study in the English population showed in fact that costal visits are often associated with lower PA-intensity as compared to visits to other natural environments, but because of longer duration costal visits can lead to greater overall energy expenditure [24]. However, it is important to consider that the extent to which one frequently engages in these activities is of course limited by whether or not one has access to the seaside or other coats [24]. This is corroborated by the regional distribution of respondents reporting to engage in activities by/on the sea fairly often, which was largest for those who live in regions with an extensive costal area (i.e., Agder and Rogaland), whereas it was quite low in the inner regions of Norway (i.e., Hedmark and Oppland). On the other hand, walking in the forest was less subjected to regional differences (see Figure S2h). Activities such as skiing and hiking in the mountains, which were previously reported to be among the most popular activities in Norway [13,19], were less endorsed in our study, especially in the LA group. This is not surprising, if one consider that such activities are in general endorsed occasionally, and are often perceived as physically demanding by many people. Hiking in the mountains was also subjected to quite large regional differences. Cycling was a somewhat frequent activity, but this seems to be more the case for those with higher PA status, whereas only a small amount of the LA group reported to frequently engage in this activity. 

Anyway, it should be noted that the implications of the finding that GE is so popular go beyond the health-benefits of PA alone: because of the exposure to nature, GE can in fact provide additional health benefits [1,4] and contribute to the population health in a broader sense also among those who do not meet the PA recommendations. Our findings show that the extent to which the respondent perceived to be frequently exposed to potentially restorative experiences of nature (i.e., whether they reported to experience nature’s quietness “fairly often”) was significantly and positively associated with the amounts of weekly GE they engage in. Interestingly, a surprisingly large amount of respondents who did not engage in weekly GE (46%) reported to frequently experience nature’s quietness. Of course individuals can be exposed to nature simply by sitting or lying in their garden or in a park, or even through views from windows in their workplace or in their homes. This might however in part depend on the limitations of our restrictive definition of “green exercise”. For example, we excluded from such definitions “active transport” and “walking the dog or other domestic animal”, which are activities that might be performed in natural environments. 

More surprising was the findings that a somewhat large amount of those who engage in high levels of weekly GE (i.e., 22% of those who engage in in GE for ≥150 min/week) reported to never/seldom experience nature’s quietness. This might suggests that not always GE is associated with restorative experiences of nature. However, it is important to take into consideration individual perceptions of what “nature’s quietness” is. It was for example reported that individuals who are occasionally engaged in more “extreme” forms of GE taking place in remote and uncontaminated nature, tent to attribute a lower restorative value to local natural environments, thought they might still enjoy using them for their weekly PA [7].

### 4.2. Green Exercise and Perceived Promoting Factors across Social Groups

GE was not evenly distributed in the population, although the findings don’t really reveal worrying inequities but rather encouraging patterns. Especially, PA is known to decrease with growing age [12], and our findings suggest that this is especially the case for forms of PA such as gym-based exercise, organized sports, active transport, and occupational PA. Thus, the fact that GE was especially popular among older adults indicates that GE is an important source of PA in these individuals, contributing to maintaining or enhancing their health profile. The fact that GE was especially popular among those who live with spouse/partner as compared with those who live alone also have possible implications in a public health perspective. PA is known to decrease after life events such as getting married [25], and GE might represent a buffer that reduces the impact of such events [26]. On the other hand, this is in line with previous findings that age-related decreases in PA are attenuated in older adults living with their spouse/partner [13].

In line with previous studies [19], we did not find a significant association of weekly GE with the respondents’ socio-economic status (education level or household income), although one may notice that the prevalence of respondents who engaged in some of the specific forms of GE tended to increase with increasing socio-economic status (see Figure S2c,d). This is a finding of interest, especially when considering that social gradients were observed in relation with other forms of PA, such as gym-based exercise and active transport. It is known that people with lower socio-economic status are less likely to engage in sufficient levels of PA [27]. On the other hand, it was previously found that socio-economic inequalities in health are less pronounced in people who have better availability of natural environments, and an explanation for this phenomenon is that such environments provide, along with other health benefits, more opportunities for engaging in PA in disadvantaged groups [28]. Altogether, this corroborates the potential of GE in fostering more equity in PA participation.

In contrast with what is suggested in some English studies [24,29,30], we found no differences of weekly GE between urban and rural areas in the overall sample. Differently, we observed that centrality has an impact on active transport and gym-based exercise. These findings are likely to depend on the fact that urban areas (especially large cities) have higher degree of mixed-land use and connectivity, whereas in Norway there is large availability of natural environments that offer opportunities to engage in GE, even in proximity of urban settlements. On the other hand, geographical determinants of weekly GE seem to be primarily linked to region of residence, possibly due to environmental factors such as the weather/climate and the type or quality of the available natural environments. Despite such differences, the levels of GE remained nevertheless quite high all-over Norway, even in northern and inner regions. 

For what concerns the perceived factors that promote GE, we found that “time flexibility” was generally perceived as the most important factors that promote GE. This was not surprising, considering that “lack of time” is known to be the most commonly reported barriers to PA [12,31] also in relation with visitation of natural environments where one may engage in GE [32]. “PA-supportive places” was also generally considered as an important factor that promotes GE, which is also in line with the literature: recent studies show in fact that the extent to which neighbor natural environments are perceived as supportive for PA, and not just the proximity to *any* natural environment, is a much more important predictor of GE [15,16]. In this study, we found however that “PA-supportive places” was perceived as more restraining by those who already have high PA levels, suggesting that improving the access to such places might not necessarily help enhancing PA levels among those who need it most, that is the LA group. 

It might be surprising that “accessibility to nature” was generally rated as little important by most respondents. As mentioned above, in Norway there is abundance of natural environments, therefore accessibility to nature might not represent a *common* barrier. Our analysis found however significant relationships of age, education levels and household income with the extent to which accessibility to nature was perceived as important factors that promotes GE. More specifically, the elderly and those with lower education level and household income assigned significantly higher ratings to this factor as compared with younger adults and those with higher education level and household income. This indicates that, although a large number of adult Norwegians have good access to natural environments, yet such accessibility is subjected to a social gradient. 

### 4.3. Strength and Limitations of the Study

This study provides a novel and unique insight into GE habits among adult Norwegians, emphasizing the potentials of this special form of PA to health equity. Strengths of this study are its population-based design, the large sample and the attention given to difference in PA levels within a regular week. The fact that different forms of PA were included also provided valuable perspectives to better understand the “GE phenomenon” in Norway and encourage further research in this field. 

The sample is well balanced for sex, age, and geographical distribution, but the respondents’ socio-economic status was somewhat higher compared to national figures. Although this gap could be partly explained by differences in the instruments used in our survey as compared with the national survey [23], such gap could have limited our ability to identify socio-economic differences with respect to weekly GE and the perceived promoting factors, and it might even have negative impact on the generalizability of our findings. Moreover, the dataset did not provide information about immigration background, which could have provided a better understanding of possible inequities in GE participation. Other limitations of our study concerns the instrument used to measure PA. First, as shown in previous studies [33], the self-reported nature of our instrument could have produced overestimated measures of PA. Moreover, in our study moderate- and vigorous-intensity PA were conflated. Similar instruments have been previously used by Norwegian national institutions [13] as well as in scientific studies (see for example Fan, Das and Chen, 2011, [34] and Librett et al., 2006, [35]). Such instrument is nevertheless likely to have underestimated PA levels for individuals who would otherwise meet WHO recommendations for *vigorous*-intensity PA (i.e., 75 min/week). Last but not least, the measures of GE used in this study might have over-lapped with other forms of PA, or even under-estimated the actual extent to which the respondents are exposed to nature whilst engaging in PA. For instance, the instrument used was unable to detect whether the respondents were exposed to nature when they engaged in activities such as commuting to work/school, walking the dog, participating in sports, etc. 

## 5. Conclusions

Green exercise is a very popular source of weekly physical activity among adult Norwegians, as well as among those with lower physical activity levels and other groups of interest to public health, such as the elderly and those with lower socio-economic status. Promotion of green exercise could be important for enhancing physical activity in the Norwegian population, and it might even foster more equity in physical activity participation. In this sense, it is important that municipalities ensure good accessibility to nature, especially to the elderly in more deprived areas. Although these results are specific for the Norwegian population, they may apply to other countries where, like in Norway, there is abundance of natural environments and where experiences in contact with nature are assigned great pedagogical and cultural value. 

## Figures and Tables

**Table 1:** Perceived factors that promote green exercise in adult Norwegians: included items and sample’s mean values.

Identifies Components and Included Items ^a^	N ^b^	Eigenvalues	α
Accessibility to nature - “Better parking opportunities by parks, green spaces and other natural environments from my homeplace” - “Better collective transport to parks …” - “Better access to parks…” - “Better accessibility to parks… for people with disabilities” - “Better information about walking/hiking trails in the vicinity”	1288	6.22	0.90
Social support - “That I am invited by friends” - “That I can be together with others” - “That I am invited from groups or associations”	1358	1.84	0.81
PA-supportive places - “Better access to cycling routes” - “Better access to ski-trails in the winter” - “Better access to walking routes” - “Better access to walking/hiking trails in the forest and open spaces” - “Better access to the seaside, beaches and coasts”	1321	1.25	0.86
Institutional support - “Whether the doctors recommends me to be more physically active” - “That the authorities carry out informational campaigns”	1345	1.01	0.51
Time flexibility ^c^ - “That I don’t need to join everytime, but that I can do it when it suits me”	1347	-	-

^a^ Factor loading, from the highest to lowest. ^b^ Rating scale: 1 = “Not important; 4 = “Very important”; different sample sizes are result of excluding respondents who answered that all the individual items “did not apply” to them. ^c^ The item was excluded from the main categories because of poor factor loading.

**Table 2:** Weekly green exercise in relation with socio-demographic characteristics in adult Norwegians (*n* = 2168).

Population Sub-Group	Total Sample (%)	Weekly Green Exercise		
		No ^a^ (Row %)	Yes ^b^ (Row %)	Unadjusted OR (95% CI)
**Sex**				
Male	50.4	39.6	60.4	-
Female	49.6	37.3	62.7	n.s.
**Age**				
Young adults (18–44 years)	30.3	48.0	52.0	-
Mid-age adults (45–64 years)	48.7	39.1	60.9	1.44 (1.18–1.75) ***
Older adults (>65 years)	21.0	23.0	77.0	3.09 (2.37–4.03) ***
**Education level**				
Below upper secondary education (≤10 years )	8.4	39.3	60.7	-
Upper secondary education (11–13 years)	28.1	39.9	60.1	n.s.
Higher education (>13 years)	46.1	36.9	63.1	n.s.
Currently studying	17.4	39.5	60.5	n.s.
**Household income (6 NOK ≈ 1 USD)**				
<399,000	27.2	40.7	59.3	-
400,000–799,000	29.3	37.3	62.7	n.s.
>800,000	33.7	35.9	64.1	n.s.
Missing	9.8	44.1	55.9	-
**Living situation (living with…)**	0.0			
Spouse or partner	69.3	35.9	64.1	-
Alone	26.1	42.4	57.6	0.76 (0.62–0.93) **
Parents or friends	4.6	54.5	45.5	0.47 (0.31–0.70) ***
**Small children at home**				
Yes	28.9	40.1	59.9	-
No	71.1	37.7	62.3	n.s.
**Centrality**				
Large city	36.0	39.8	60.2	-
Small city	24.1	36.4	63.6	n.s.
Small town/Village	24.2	40.0	60.0	n.s.
Countryside	15.7	35.9	64.1	n.s.
**Region**				
Oslo and Akershus	30.6	41.0	59.0	-
Hedmark and Oppland	8.1	41.7	58.3	n.s.
South Eastern Norway	13.8	41.0	59.0	n.s.
Agder and Rogaland	11.7	33.2	66.8	1.40 (1.03–1.90) *
Western Norway	17.8	34.5	65.5	1.32 (1.02–1.71) *
Trøndelag	8.4	33.5	66.5	n.s.
Northern Norway	9.7	41.4	58.6	n.s.

^a^ Reporting not to engage in green exercise (0 min/week). ^b^ Reporting to engage in green exercise for ≥1 min/week. Statistics refer to odd ratio (OR) and 95% confidence interval (CI) with respect with the first category. n.s. = Non-significant, i.e., *p* ≥ 0.005; * *p* < 0.05; ** *p* < 0.01; *** *p* < 0.001.

**Table 3:** Perceived factors that promote green exercise ^a^, across groups with different physical activity profile and sociodemographic characteristics in a sample of adult Norwegians (*n* = 1256 ^a^).

Population Sub-Group (*n* Percent)	Perceived Factors That Promote Green Exercise (M ± SD)				
	Accessibility to Nature	Social Support	PA Supportive Places	Institutional Support	Time Flexibility
**Weekly green exercise**					
No (34%)	2.01 ± 0.86	2.22 ± 0.70	2.27 ± 0.81	2.09 ± 0.68	2.95 ± 0.80
Yes (66%)	1.98 ± 0.81	2.17 ± 0.64	2.30 ± 0.74	2.13 ± 0.67	3.00 ± 0.74
*MANOVA: n.s.*					
*ANOVA* ^b^	-	-	-	-	-
**PA status**					
LA (40%)	2.00 ± 0.85	2.14 ± 0.68	2.23 ± 0.78	2.11 ± 0.66	3.03 ± 0.71
RA (32%)	1.98 ± 0.79	2.22 ± 0.63	2.30 ± 0.74	2.09 ± 0.65	2.99 ± 0.75
HA (28%)	2.00 ± 0.84	2.20 ± 0.67	2.36 ± 0.77	2.14 ± 0.72	2.91 ± 0.83
*MANOVA: λ* = *0.98*; *F*_(*10, 2498*)_ = 2.40 **					
*ANOVA: F*_(*2, 1253*)_ = …	*n.s.*	*n.s.*	3.22 *	*n.s.*	*n.s.*
**Sex**					
Male (50%)	1.91 ± 0.80	2.12 ± 0.65	2.25 ± 0.77	2.08 ± 0.68	2.90 ± 0.79
Female (50%)	2.08 ± 0.84	2.25 ± 0.67	2.33 ± 0.76	2.15 ± 0.67	3.07 ± 0.71
*MANOVA: λ = 0.98*; *F*_(*5, 1250*)_ = 5.96 ***					
*ANOVA: F*_(*1, 1254*)_ = …	13.15 ***	12.74 ***	*n.s.*	3.94 *	17.39 ***
**Age group**					
Young adults, 18–44 years (27%)	1.95 ± 0.79	2.33 ± 0.63	2.28 ± 0.78	1.94 ± 0.68	2.97 ± 0.80
Mid-age adults, 45–64 years (52%)	1.96 ± 0.81	2.16 ± 0.67	2.28 ± 0.76	2.14 ± 0.66	3.00 ± 0.74
Older adults, >65 years (21%)	2.14 ± 0.89	2.05 ± 0.66	2.33 ± 0.75	2.29 ± 0.66	2.95 ± 0.75
*MANOVA: λ = 0.92*; *F*_(*10, 2498*)_ = 11.15 ***					
*ANOVA: F*_(*2, 1253*)_ = …	5.42 **	14.15 ***	*n.s.*	21.05 ***	*n.s.*
**Educational level**					
Below upper secondary education (8%)	2.31 ± 0.90	2.11 ± 0.65	2.37 ± 0.74	2.30 ± 0.74	3.05 ± 0.72
Upper secondary education (27%)	2.04 ± 0.87	2.15 ± 0.67	2.21 ± 0.78	2.14 ± 0.68	3.02 ± 0.80
Higher education (47%)	1.95 ± 0.77	2.21 ± 0.64	2.29 ± 0.73	2.11 ± 0.64	2.97 ± 0.73
Currently studying (18%)	1.88 ± 0.84	2.22 ± 0.71	2.37 ± 0.83	2.01 ± 0.70	2.93 ± 0.79
*MANOVA: λ = 0.95*; *F*_(*15, 3445*)_ = 4.77 **					
*ANOVA: F*_(*3, 1252*)_ = …	7.38 ***	*n.s.*	*n.s.*	4.82 **	*n.s.*
**Household income, 6 NOK ≈ 1 USD (*n* = 1151 ^c^)**					
<399,000 (28%)	2.09 ± 0.85	2.20 ± 0.68	2.32 ± 0.77	2.10 ± 0.69	2.98 ± 0.81
400,000–799,000 (33%)	2.02 ± 0.84	2.19 ± 0.68	2.27 ± 0.75	2.17 ± 0.65	2.97 ± 0.74
>800,000 (39%)	1.86 ± 0.76	2.15 ± 0.64	2.27 ± 0.77	2.06 ± 0.66	2.96 ± 0.75
*MANOVA: λ = 0.98*; *F*_(*10, 2286*)_ = 2.74 **					
*ANOVA: F*_(*2, 1148*)_ = …	8.02 ***	*n.s.*	*n.s.*	*n.s.*	*n.s.*
**Living situation**					
Spouse or partner (72%)	1.99 ± 0.83	2.16 ± 0.66	2.31 ± 0.76	2.14 ± 0.67	2.97 ± 0.75
Alone (25%)	2.01 ± 0.83	2.24 ± 0.67	2.24 ± 0.78	2.06 ± 0.69	3.02 ± 0.76
Parents or friends (4%)	1.94 ± 0.76	2.38 ± 0.65	2.30 ± 0.73	1.98 ± 0.73	2.89 ± 0.96
*MANOVA: λ = 0.98*; *F*_(*10*, *2498*)_ = 2.68 **					
*ANOVA: F*_(*2, 1153*)_ = …	-	3.92 *	-	-	-
**Small children at home**					
Yes (29%)	1.94 ± 0.80	2.21 ± 0.65	2.29 ± 0.77	2.09 ± 0.65	2.97 ± 0.74
No (71%)	2.01 ± 0.84	2.18 ± 0.67	2.29 ± 0.76	2.13 ± 0.69	2.99 ± 0.77
*MANOVA: n.s.*					
*ANOVA* ^b^	-	-	-	-	-
**Centrality**					
Large city (34%)	2.01 ± 0.83	2.18 ± 0.65	2.27 ± 0.76	2.06 ± 0.68	2.98 ± 0.75
Small city (25%)	2.05 ± 0.84	2.22 ± 0.64	2.35 ± 0.78	2.12 ± 0.69	2.97 ± 0.74
Small town/village (25%)	1.97 ± 0.83	2.18 ± 0.71	2.30 ± 0.78	2.15 ± 0.68	2.99 ± 0.77
Country-side (16%)	1.92 ± 0.79	2.15 ± 0.63	2.23 ± 0.72	2.18 ± 0.64	2.99 ± 0.79
*MANOVA: n.s.*					
*ANOVA* ^b^	-	-	-	-	-
**Region**					
Oslo and Akershus (29%)	1.98 ± 0.81	2.13 ± 0.64	2.31 ± 0.76	2.06 ± 0.66	2.97 ± 0.75
Hedmark and Oppland (9%)	1.90 ± 0.82	2.05 ± 0.66	2.30 ± 0.82	2.13 ± 0.71	2.97 ± 0.78
South Eastern Norway (13%)	1.98 ± 0.80	2.10 ± 0.70	2.29 ± 0.79	2.11 ± 0.66	3.04 ± 0.80
Agder and Rogaland (12%)	2.08 ± 0.91	2.31 ± 0.71	2.28 ± 0.79	2.24 ± 0.69	3.01 ± 0.83
Western Norway (18%)	2.01 ± 0.85	2.25 ± 0.64	2.28 ± 0.79	2.13 ± 0.68	3.02 ± 0.69
Trøndelag (9%)	1.83 ± 0.73	2.14 ± 0.59	2.15 ± 0.68	2.00 ± 0.63	2.81 ± 0.82
Northern Norway (10%)	2.11 ± 0.85	2.32 ± 0.67	2.39 ± 0.70	2.18 ± 0.71	2.99 ± 0.67
*MANOVA: λ* = *0.96*; *F*_(*30, 4982*)_ = 1.63 *					
*ANOVA: F*_(*6, 1249*)_ = …	*n.s.*	3.81 **	*n.s.*	*n.s.*	*n.s.*

^a^ The analyses are performed on a sub-sample of respondents, i.e., all those who reported future intent for green exercise; ^b^ ANOVA was not performed because significance was not achieved in the multivariate test; ^c^ The smaller sample size is result of excluding respondents who answered that they “don’t know” what is their household income or “don’t want to answer”. PA status: LA = Low PA levels (<150 min/week); RA = Recommended PA levels (150–299 min/week); HA = High PA levels (≥300 min/week). * *p* < 0.05; ** *p* < 0.01; *** *p* < 0.001.

## Supplementary Materials

The following are available online at www.mdpi.com/1660-4601/13/11/1165/s1, Figure S1: Prevalence of adult Norwegians engaging in weekly green exercise and other forms of physical activity by (*n* = 2168): (a) sex; (b) age group; (c) education level; (d) household income; (e) living situation; (f) having/not having young children in the household; (g) centrality; (h) region. Figure S2: Prevalence of adult Norwegians engaging in different dorms of green exercise “fairly often” by (*n* = 2168): (a) sex; (b) age group; (c) education level; (d) household income; (e) living situation; (f) having/not having young children in the household; (g) centrality; (h) region. Table S1: Weekly green exercise in relation with socio-demographic characteristics restricted to women (*n* = 1076); Table S2: Weekly green exercise in relation with socio-demographic characteristics restricted to older (age ≥ 65 years; *n* = 456); Table S3: Perceived factors that promote green exercise, across groups with different physical activity profile and sociodemographic characteristics restricted to women (*n* = 613); Table S4: Perceived factors that promote green exercise, across groups with different physical activity profile and sociodemographic characteristics restricted to older adult (age ≥ 65 years; *n* = 263).
