# Supplementary material for: Is Green Exercise for All? A Descriptive Study of Green Exercise Habits and Promoting Factors in Adult Norwegians
Source: Int J Environ Res Public Health. 2016 Nov 23;13(11):1165. doi: 10.3390/ijerph13111165 (PMC5129375; doi:10.3390/ijerph13111165)
Supplement: Supplementary file 1 [file ijerph-13-01165-s001.pdf]

Supplementary Materials: Is Green Exercise for All? A Descriptive Study of Green Exercise Habits and Promoting Factors in Adult Norwegians

Giovanna Calogiuri, Grete G Patil and Geir Aamodt

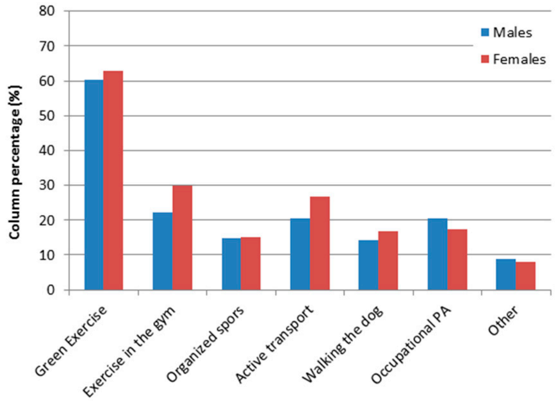

(**a**)

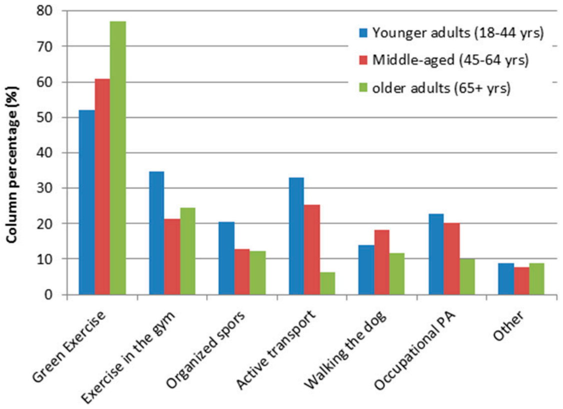

(**b**)

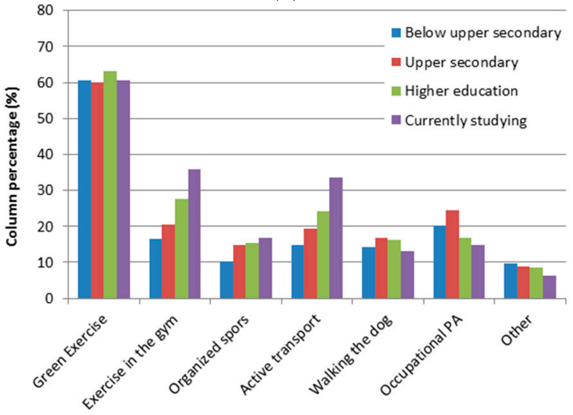

(**c**)

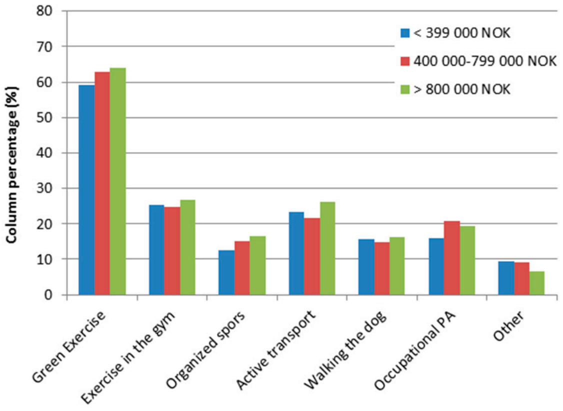

(**d**)

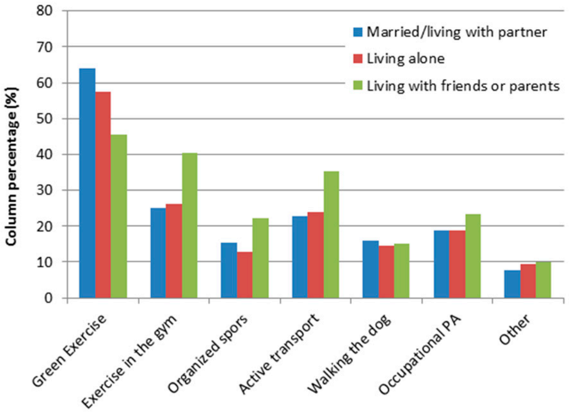

(**e**)

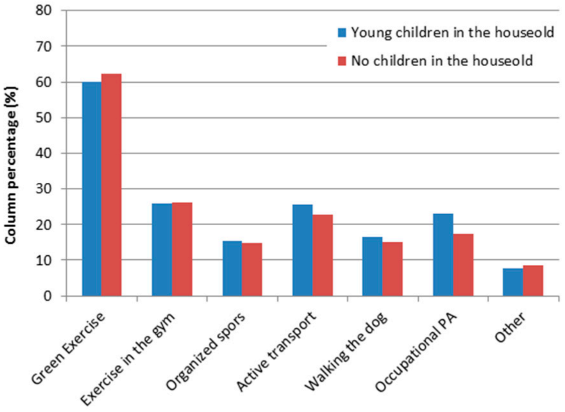

(**f**)

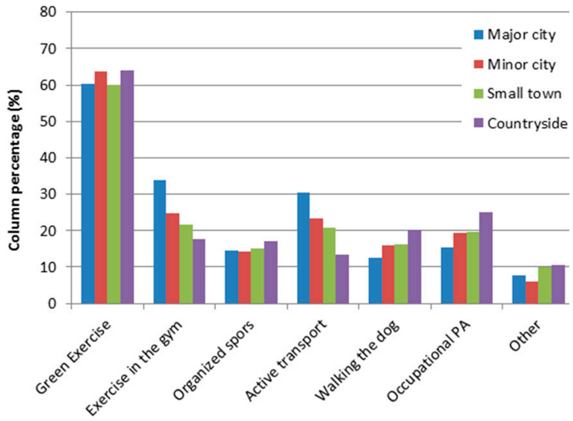

(**g**)

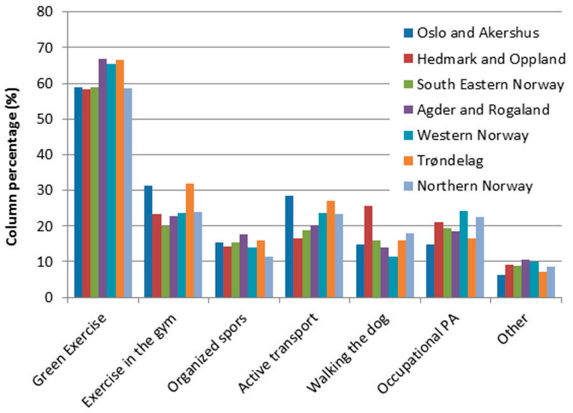

(**h**)

Figure S1. Prevalence of adult Norwegians engaging in weekly green exercise and other forms of physical activity by (n = 2168): (**a**) sex; (**b**) age group; (**c**) education level; (**d**) household income; (**e**) living situation; (**f**) having/not having young children in the household; (**g**) centrality; (**h**) region. The bars represent the prevalence of individual who reported to engage in green exercise or other forms of PA for ≥1 min/week, whereas those who reported not to engage at (=0 min/week) are not reported because it would be redundant.

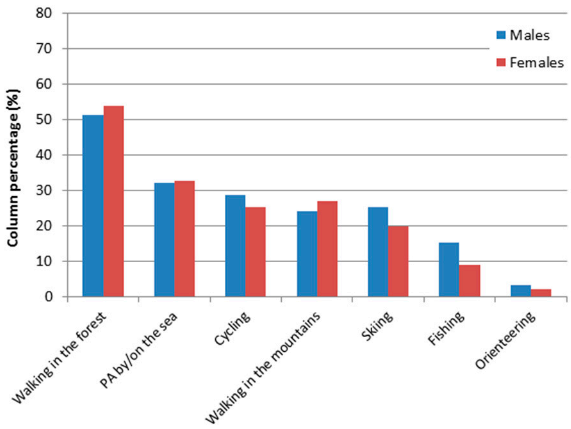

(**a**)

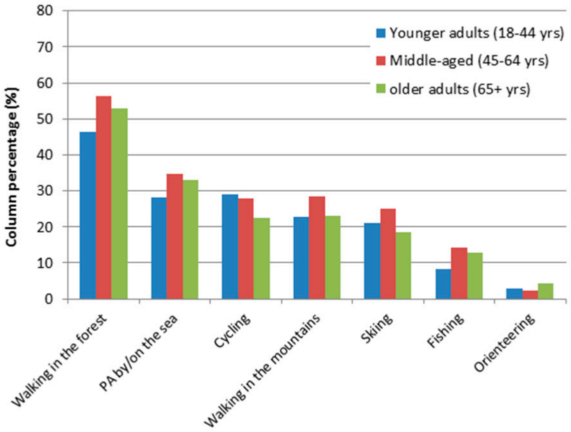

(**b**)

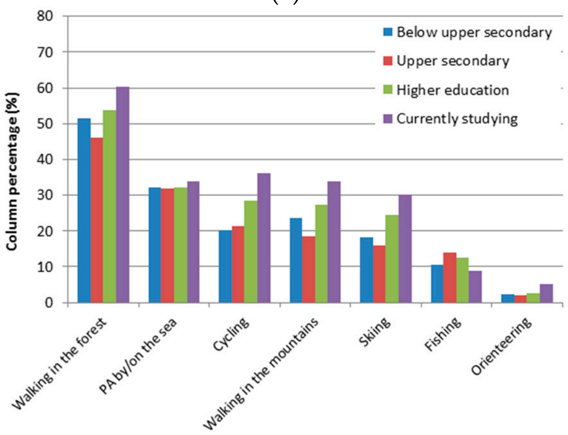

(**c**)

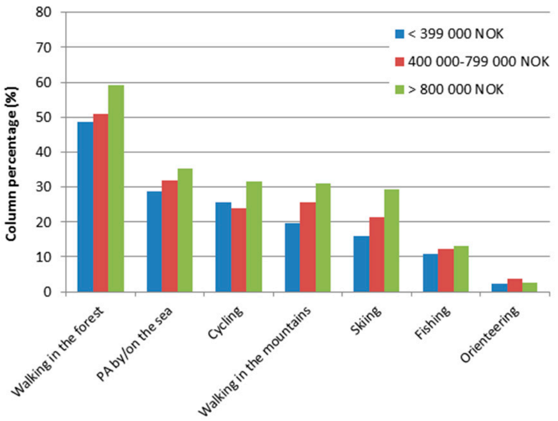

(**d**)

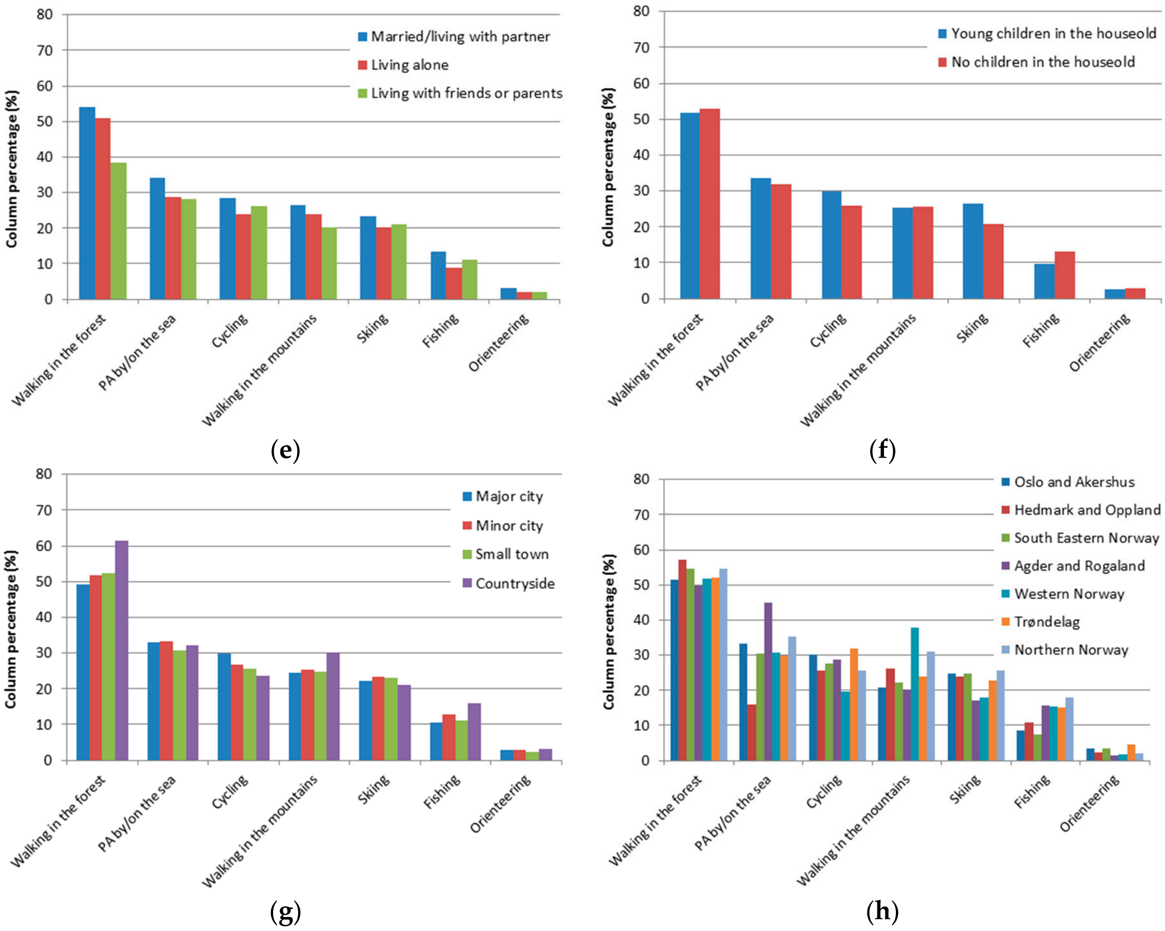

Figure S2. Prevalence of adult Norwegians engaging in different dorms of green exercise "fairly often" by (n = 2168): (**a**) sex; (**b**) age group; (**c**) education level; (**d**) household income; (**e**) living situation; (**f**) having/not having young children in the household; (**g**) centrality; (**h**) region. The bars represent the prevalence of individual who reported to engage in the specific form of green exercise "fairly often", whereas those who engaged in the activity "never/seldom" are not reported because it would be redundant.

Table S1. Weekly green exercise in relation with socio-demographic characteristics restricted to women (n = 1076).

Population Sub-Group	Total Sample (%)	Weekly Green Exercise		
		No [a] (Row %)	Yes [b] (Row %)	Unadjusted OR (95% CI)
**Age**				
Young adults (18–44 years)	32.4	39.2	28.4	-
Mid-age adults (4–64 years)	49.0	49.6	48.6	1.35 (1.02–1.77) *
Older adults (>65 years)	18.6	11.2	23.0	2.82 (1.90–4.17) ***
**Education level**				
Below upper secondary education (≤10 years)	8.0	7.0	8.6	-
Upper secondary education (1–13 years)	29.7	31.4	28.7	n.s.
Higher education (>13 years)	48.3	46.6	49.3	n.s.
Currently studying	13.9	15.0	13.3	n.s.
**Household income (6 NOK ≈ 1 USD)**				
<399,000	35.8	38.5	34.2	-
400,000–799,000	30.6	31.0	30.3	n.s.
>800,000	33.6	30.5	35.5	n.s.
Missing	11.9	-	-	
**Living situation (living with…)**				
Spouse or partner	64.6	61.3	66.5	-

Alone	30.6	32.2	29.6	n.s.
Parents or friends	4.8	6.5	3.9	0.55 (0.3–0.96) *
**Small children at home**				
Yes	28.6	28.7	28.6	-
No	71.4	71.3	71.4	n.s.
**Centrality**				
Large city	35.4	36.7	34.7	-
Small city	25.7	24.7	26.2	n.s.
Small town/Village	23.2	24.9	22.2	n.s.
Countryside	15.7	13.7	16.9	n.s.
**Region**				
Oslo and Akershus	30.0	34.7	27.3	-
Hedmark and Oppland	7.4	8.7	6.7	n.s.
South Eastern Norway	14.2	14.7	13.9	n.s.
Agder and Rogaland	12.0	10.0	13.2	1.68 (1.0–2.59) *
Western Norway	18.3	15.2	20.1	1.68 (1.1–2.45) **
Trøndelag	8.9	7.2	9.9	1.75 (1.0–2.84) *
Northern Norway	9.1	9.5	8.9	n.s.

[a] Reporting not to engage in green exercise (0 min/week); [b] Reporting to engage in green exercise for ≥1 min/week. Statistics refer to odd ratio (OR) and 95% confidence interval (CI) with respect with the first category. * $p < 0.05$; ** $p < 0.01$; *** $p < 0.001$.

Table S2. Weekly green exercise in relation with socio-demographic characteristics restricted to older (age ≥ 65 years; $n = 456$).

Population Sub-Group	Total Sample (%)	Weekly Green Exercise		
		No [a] (Row %)	Yes [b] (Row %)	Unadjusted OR (95% CI)
**Gender**				
Male	56.1	57.1	55.8	-
Female	43.9	42.9	44.2	n.s.
**Education level**				
Below upper secondary education (≤10 years)	17.5	20.0	16.8	-
Upper secondary education (11–13 years)	20.4	19.0	20.8	n.s.
Higher education (>13 years)	42.8	39.0	43.9	n.s.
Currently studying	19.3	21.9	18.5	n.s.
**Household income (6 NOK ≈ 1 USD)**				
<399,000	35.4	37.4	34.8	-
400,000–799,000	44.5	45.1	44.3	n.s.
>800,000	20.1	17.6	20.9	n.s.
Missing	10.7	-	-	-
**Living situation (living with…)**				
Spouse or partner	75.9	77.1	75.5	-
Alone	23.7	1.0	24.2	n.s.
Parents or friends	0.4	21.9	0.3	n.s.
**Small children at home**				
Yes	4.2	2.9	4.6	-
No	95.8	97.1	95.4	n.s.
**Centrality**				
Large city	31.8	40.0	29.3	-
Small city	24.8	21.0	25.9	n.s.
Small town/Village	24.3	26.7	23.6	n.s.
Countryside	19.1	12.4	21.1	2.32 (1.16–4.63) *
**Region**				
Oslo and Akershus	30.0	31.4	29.6	-
Hedmark and Oppland	9.0	8.6	9.1	n.s.
South Eastern Norway	14.5	16.2	14.0	n.s.
Agder and Rogaland	12.1	11.4	12.3	n.s.

Western Norway	16.4	17.1	16.2	n.s.
Trøndelag	9.0	6.7	9.7	n.s.
Northern Norway	9.0	8.6	9.1	n.s.

[a] Reporting not to engage in green exercise (0 min/week); [b] Reporting to engage in green exercise for ≥1 min/week. Statistics refer to odd ratio (OR) and 95% confidence interval (CI) with respect with the first category. * $p < 0.05$.

Table S3. Perceived factors that promote green exercise [a], across groups with different physical activity profile and sociodemographic characteristics restricted to women (n = 613 [a]).

Population Sub-Group (*n*)	Perceived Factors That Promote Green Exercise (M ± SD)				
	Accessibility to Nature	Social Support	PA Supportive Places	Institutional Support	Time Flexibility
**Weekly green exercise**					
No (33%)	2.14 ± 0.86	2.24 ± 0.70	2.31 ± 0.80	2.12 ± 0.70	3.06 ± 0.72
Yes (67%)	2.06 ± 0.85	2.25 ± 0.67	2.35 ± 0.75	2.16 ± 0.68	3.07 ± 0.71
MANOVA: n.s.					
ANOVA [b]	-	-	-	-	-
**PA status**					
LA (40%)	2.13 ± 0.88	2.27 ± 0.69	2.26 ± 0.78	2.13 ± 0.68	3.10 ± 0.67
RA (34%)	2.07 ± 0.83	2.26 ± 0.65	2.35 ± 0.74	2.14 ± 0.69	3.07 ± 0.72
HA (27%)	2.04 ± 0.83	2.19 ± 0.69	2.44 ± 0.76	2.18 ± 0.70	3.01 ± 0.77
MANOVA: $\lambda = 0.96$; $F_{(10, 1212)} = 2.56$ *					
ANOVA: $F_{(1, 610)} = \ldots$	*n.s.*	*n.s.*	*4.43 **	*n.s.*	*n.s.*
**Age group**					
Young adults, 18–44 years (30%)	2.06 ± 0.82	2.33 ± 0.63	2.34 ± 0.76	1.98 ± 0.71	3.04 ± 0.76
Mid-age adults, 45–64 years (52%)	2.05 ± 0.83	2.24 ± 0.69	2.32 ± 0.76	2.19 ± 0.68	3.07 ± 0.68
Older adults, >65 years (18%)	2.22 ± 0.96	2.15 ± 0.70	2.38 ± 0.79	2.31 ± 0.61	3.10 ± 0.72
MANOVA: $\lambda = 0.94$; $F_{(10, 1212)} = 3.65$ ***					
ANOVA: $F_{(2, 610)} = \ldots$	*n.s.*	*n.s.*	*n.s.*	*7.36 ***	*n.s.*
**Educational level**					
Below upper secondary education (7%)	2.42 ± 0.94	2.15 ± 0.70	2.30 ± 0.78	2.28 ± 0.79	3.13 ± 0.78
Upper secondary education (28%)	2.15 ± 0.94	2.24 ± 0.69	2.29 ± 0.77	2.19 ± 0.72	3.16 ± 0.71
Higher education (51%)	2.04 ± 0.77	2.26 ± 0.65	2.33 ± 0.74	2.14 ± 0.65	3.03 ± 0.68
Currently studying (14%)	1.97 ± 0.87	2.26 ± 0.72	2.48 ± 0.82	2.02 ± 0.69	2.97 ± 0.77
MANOVA: $\lambda = 0.93$; $F_{(15, 1671)} = 2.91$ ***					
ANOVA: $F_{(3, 609)} = \ldots$	*3.43 **	*n.s*	*n.s*	*n.s*	*n.s*
**Household income, 6 NOK ≈ 1 US$ (*n* = 554 [c])**					
<399,000 (33%)	2.19 ± 0.84	2.28 ± 0.70	2.39 ± 0.74	2.16 ± 0.71	3.06 ± 0.77
400,000–799,000 (32%)	2.07 ± 0.86	2.25 ± 0.70	2.34 ± 0.75	2.18 ± 0.65	3.04 ± 0.71
>800,000 (35%)	1.93 ± 0.79	2.19 ± 0.62	2.32 ± 0.79	2.07 ± 0.67	3.03 ± 0.68
MANOVA: n.s.					
ANOVA [b]	-	-	-	-	-
**Living situation**					
Spouse or partner (66%)	2.07 ± 0.86	2.22 ± 0.67	2.34 ± 0.77	2.17 ± 0.68	3.05 ± 0.68
Alone (30%)	2.12 ± 0.83	2.29 ± 0.69	2.34 ± 0.75	2.13 ± 0.70	3.13 ± 0.72
Parents or friends (4%)	2.06 ± 0.84	2.48 ± 0.68	2.29 ± 0.72	1.95 ± 0.71	2.89 ± 1.03
MANOVA: n.s.					
ANOVA [b]	-	-	-	-	-
**Small children at home**					
Yes (29%)	1.98 ± 0.79	2.23 ± 0.65	2.30 ± 0.74	2.09 ± 0.67	3.02 ± 0.69
No (71%)	2.13 ± 0.87	2.26 ± 0.69	2.35 ± 0.77	2.17 ± 0.70	3.08 ± 0.72
MANOVA: n.s.					
ANOVA [b]	-	-	-	-	-
**Centrality**					
Large city (35%)	2.07 ± 0.85	2.24 ± 0.68	2.29 ± 0.73	2.1 ± 0.69	3.11 ± 0.65
Small city (27%)	2.15 ± 0.84	2.31 ± 0.68	2.47 ± 0.79	2.17 ± 0.70	3.08 ± 0.74
Small town/village (23%)	2.08 ± 0.87	2.25 ± 0.68	2.30 ± 0.79	2.20 ± 0.68	2.99 ± 0.72
Country-side (16%)	2.00 ± 0.84	2.15 ± 0.64	2.27 ± 0.74	2.14 ± 0.69	3.05 ± 0.79
MANOVA: n.s.					
ANOVA [b]	-	-	-	-	-
**Region (1256)**					
Oslo and Akershus (27%)	2.05 ± 0.85	2.17 ± 0.67	2.27 ± 0.77	2.09 ± 0.65	3.01 ± 0.71

Hedmark and Oppland (8%)	2.04 ± 0.97	2.07 ± 0.66	2.21 ± 0.91	2.05 ± 0.74	2.98 ± 0.77
South Eastern Norway (15%)	2.01 ± 0.77	2.28 ± 0.66	2.37 ± 0.75	2.11 ± 0.68	3.14 ± 0.65
Agder and Rogaland (14%)	2.21 ± 0.9	2.43 ± 0.68	2.46 ± 0.78	2.30 ± 0.69	3.11 ± 0.76
Western Norway (18%)	2.12 ± 0.88	2.25 ± 0.66	2.34 ± 0.78	2.17 ± 0.71	3.09 ± 0.64
Trøndelag (9%)	1.9 ± 0.76	2.20 ± 0.67	2.20 ± 0.68	2.07 ± 0.62	3.07 ± 0.79
Northern Norway (10)	2.25 ± 0.79	2.34 ± 0.72	2.52 ± 0.63	2.26 ± 0.74	3.08 ± 0.76
MANOVA: n.s.					
ANOVA [b]	-	-	-	-	-

[a] The analyses are performed on a sub-sample of respondents, i.e., all women who reported future intent for green exercise; [b] ANOVA was not performed because significance was not achieved in the multivariate test; [c] The smaller sample size is result of excluding respondents who answered that they "don't know" what is their household income or "don't want to answer". PA status: LA = Low PA levels (<150 min/week); RA = Recommended PA levels (150–299 min/week); HA = High PA levels (≥300 min/week). * $p < 0.05$; ** $p < 0.01$; *** $p < 0.001$

Table S4. Perceived factors that promote green exercise [a], across groups with different physical activity profile and sociodemographic characteristics restricted to older adult (age ≥ 65 years; $n = 263$ [a]).

**Population Sub-Group**	**Perceived Factors That Promote Green Exercise (M ± SD)**				
	**Accessibility to Nature**	**Social Support**	**PA Supportive Places**	**Institutional Support**	**Time Flexibility**
**Weekly green exercise**					
No (19%)	2.23 ± 0.98	1.92 ± 0.74	2.36 ± 0.73	2.35 ± 0.70	3.02 ± 0.84
Yes (81%)	2.11 ± 0.87	2.08 ± 0.66	2.33 ± 0.78	2.27 ± 0.66	2.94 ± 0.75
MANOVA: n.s.					
ANOVA [b]	-	-	-	-	-
**PA status**					
LA (38%)	2.16 ± 0.90	1.99 ± 0.72	2.39 ± 0.79	2.28 ± 0.67	2.96 ± 0.77
RA (32%)	2.11 ± 0.91	2.11 ± 0.66	2.25 ± 0.76	2.23 ± 0.65	2.95 ± 0.72
HA (31%)	2.12 ± 0.86	2.05 ± 0.65	2.35 ± 0.76	2.33 ± 0.69	2.95 ± 0.82
MANOVA: n.s.					
ANOVA [b]	-	-	-	-	-
**Sex**					
Male (58%)	2.07 ± 0.83	1.98 ± 0.65	2.30 ± 0.76	2.26 ± 0.71	2.85 ± 0.78
Female (42%)	2.22 ± 0.96	2.15 ± 0.70	2.38 ± 0.79	2.31 ± 0.61	3.10 ± 0.72
MANOVA: $\lambda = 0.95$; $F_{(5, 257)} = 2.74$ *					
ANOVA: $F_{(1, 261)} = \ldots$	*n.s.*	*4.41 **	*n.s.*	*n.s.*	*9.64 ***
**Educational level**					
Below upper secondary education (18%)	2.51 ± 0.88	2.16 ± 0.63	2.46 ± 0.66	2.57 ± 0.69	3.06 ± 0.73
Upper secondary education (21%)	2.19 ± 0.89	2.06 ± 0.70	2.40 ± 0.80	2.28 ± 0.64	3.07 ± 0.80
Higher education (42%)	2.01 ± 0.84	2.03 ± 0.67	2.23 ± 0.77	2.19 ± 0.62	2.85 ± 0.77
Currently studying (19%)	1.98 ± 0.90	1.98 ± 0.71	2.36 ± 0.81	2.21 ± 0.74	2.96 ± 0.74
MANOVA: $\lambda = 0.90$; $F_{(15, 704)} = 1.78$ *					
ANOVA: $F_{(3, 259)} = \ldots$	*3.88 ***	*n.s.*	*n.s.*	*3.82 ***	*n.s.*
**Household income, 6 NOK ≈ 1 US$) ($n = 239$ [c])**					
<399,000 (34%)	2.33 ± 0.90	2.09 ± 0.74	2.48 ± 0.77	2.37 ± 0.68	2.91 ± 0.82
400,000–799,000 (45%)	2.05 ± 0.84	2.01 ± 0.64	2.23 ± 0.75	2.26 ± 0.61	2.95 ± 0.71
>800,000 (21%)	1.84 ± 0.84	2.03 ± 0.68	2.20 ± 0.74	2.07 ± 0.64	2.95 ± 0.83
MANOVA: n.s.					
ANOVA [b]	-	-	-	-	-
**Living situation**					
Spouse or partner (79%)	2.07 ± 0.89	2.01 ± 0.66	2.31 ± 0.77	2.27 ± 0.67	2.93 ± 0.76
Alone (21%)	2.37 ± 0.84	2.20 ± 0.74	2.42 ± 0.74	2.34 ± 0.68	3.05 ± 0.77
Parents or friends (0%)	-	-	-	-	-
MANOVA: n.s.					
ANOVA [b]	-	-	-	-	-
**Small children at home**					
Yes (3%)	1.97 ± 0.59	1.87 ± 0.63	2.17 ± 0.68	2.20 ± 0.54	2.90 ± 1.10
No (97%)	2.14 ± 0.90	2.06 ± 0.68	2.34 ± 0.77	2.28 ± 0.67	2.96 ± 0.75
MANOVA: n.s.					
ANOVA [b]	-	-	-	-	-
**Centrality**					
Large city (34%)	2.12 ± 0.92	2.02 ± 0.69	2.29 ± 0.78	2.29 ± 0.70	3.02 ± 0.78

Small city (22%)	2.17 ± 0.90	2.03 ± 0.68	2.48 ± 0.80	2.30 ± 0.74	2.90 ± 0.78
Small town/village (25%)	2.24 ± 0.93	2.07 ± 0.73	2.37 ± 0.76	2.25 ± 0.67	2.90 ± 0.76
Country-side (19%)	1.98 ± 0.76	2.10 ± 0.61	2.18 ± 0.70	2.28 ± 0.55	2.96 ± 0.74
MANOVA: n.s.					
ANOVA [b]	-	-	-	-	-
**Region**					
Oslo and Akershus (30%)	2.14 ± 0.86	2.07 ± 0.73	2.27 ± 0.77	2.28 ± 0.67	3.02 ± 0.73
Hedmark and Oppland (9%)	2.06 ± 0.70	1.88 ± 0.59	2.47 ± 0.80	2.35 ± 0.66	2.85 ± 0.72
South Eastern Norway (18%)	2.12 ± 0.84	2.11 ± 0.73	2.39 ± 0.79	2.19 ± 0.75	3.08 ± 0.84
Agder and Rogaland (10%)	2.24 ± 0.99	2.13 ± 0.74	2.33 ± 0.77	2.20 ± 0.71	2.68 ± 0.98
Western Norway (17%)	2.14 ± 1.02	2.04 ± 0.63	2.26 ± 0.80	2.29 ± 0.68	2.98 ± 0.68
Trøndelag (10%)	2.00 ± 0.88	1.94 ± 0.53	2.34 ± 0.81	2.34 ± 0.61	2.93 ± 0.78
Northern Norway (10%)	2.19 ± 0.94	2.09 ± 0.70	2.42 ± 0.66	2.34 ± 0.61	2.93 ± 0.68
MANOVA: n.s.					
ANOVA [b]	-	-	-	-	-

[a] The analyses are performed on a sub-sample of respondents, i.e., all those who reported future intent for green exercise; [b] ANOVA was not performed because significance was not achieved in the multivariate test; [c] The smaller sample size is result of excluding respondents who answered that they "don't know" what is their household income or "don't want to answer". PA status: LA = Low PA levels (<150 min/week); RA = Recommended PA levels (150–299 min/week); HA = High PA levels (≥300 min/week). * $p < 0.05$; ** $p < 0.01$.

© 2016 by the authors. Submitted for possible open access publication under the terms and conditions of the Creative Commons Attribution (CC-BY) license (http://creativecommons.org/licenses/by/4.0/).
